# Development of Colorimetric Whole-Cell Biosensor for Detection of Heavy Metals in Environment for Public Health

**DOI:** 10.3390/ijerph182312721

**Published:** 2021-12-02

**Authors:** Yihyang Kim, Hyeunseok Choi, Weon Ho Shin, Jong-Min Oh, Sang-Mo Koo, Younghun Kim, Taek Lee, Byung Jo Yu, Chulhwan Park

**Affiliations:** 1 Department of Chemical Engineering, Kwangwoon University, Seoul 01897, Korea; kyscent@nate.com (Y.K.); korea1@kw.ac.kr (Y.K.); 2 Eco-friendly Convergence Materials Research Part, Korea Institute of Industrial Technology (KITECH), Cheonan 31056, Korea; hchoi@kitech.re.kr; 3 Department of Electronic Materials Engineering, Kwangwoon University, Seoul 01897, Korea; weonho@kw.ac.kr (W.H.S.); jmOH@kw.ac.kr (J.-M.O.); smkoo@kw.ac.kr (S.-M.K.)

**Keywords:** heavy metal, whole-cell biosensor, promoter, reporter gene, bioluminescence, red fluorescent protein, immobilization, public health

## Abstract

Heavy metals cause various fetal diseases in humans. Heavy metals from factory wastewater can contaminate drinking water, fish, and crops. Inductively coupled plasma-mass spectrometry (ICP-MS) and atomic absorption spectrometry (AAS) are commonly used to analyze heavy metal contents; however, these methods require pre-treatment processes and are expensive and complex. To overcome these limitations, three metal-sensing materials using a whole-cell biosensor in *Escherichia coli* (*E. coli*) were developed. Strains were engineered to harbor three kinds of plasmids containing the *copA*, *zntA*, and *mer* promoters for sensing copper, cadmium, and mercury, respectively. The luciferase (*lux*) gene was inserted as a reporter into the plasmid, which was later replaced with a fused protein sequence containing OmpA (1–159) and mCherry for optical detection. The constructed strains could detect mercury, cadmium, and copper at 0.1–0.75 ppm, 0.2–0.75 ppm, and 2–7.5 ppm, respectively, with linearity values of 0.99030, 0.99676, and 0.95933, respectively. The immobilization linearity value was 0.99765. Notably, these three heavy metals could be detected by visual analysis of the strains. Overall, these findings establish this novel sensor as a potential approach for heavy metal detection in biological samples and foods.

## 1. Introduction

Heavy metals, including cadmium and mercury, are metals with atomic masses between those of copper and lead. With industrial development, environmental heavy metal contamination is becoming a serious problem and requires urgent remediation [1]. For example, heavy metals were detected in the soil of woodlands, grasslands, and farmlands in Suohuangcang National Wetland Park [2]. This kind of contamination can affect food, like fish, crops, and water. In Russia, residents of Chukotka were exposed to persistent mercury contamination in foods [3]. Exposure to heavy metal-contaminated foods damages human health [3,4]. For example, cadmium and lead cause permanent damage to the central nervous system or death. Mercury and arsenic can cause Guillain–Barre syndrome or death [5]. Among the various kinds of contaminated foods, fish products typically have the highest concentration of heavy metals [6]. Therefore, simple and low-cost approaches for heavy metal detection are urgently required. Conventional methods of heavy metal measurement are inductively coupled with plasma-mass spectrometry (ICP-MS) and atomic absorption spectrometry (AAS). However, these methods require laborious sample pre-treatment (e.g., decomposition of nitric acid at a high temperature), expensive instruments, and skilled technicians [6,7]. Thus, these approaches are not practical for the detection of heavy metals in food products. The nanoparticle sensors for heavy metal detection are unreliable when the heavy metal concentrations are below a certain level (0.6–1 ppm); selectivity issues arise at very low heavy metal concentrations and the target metal cannot be distinguished from other metals [8]. Therefore, a whole-cell biosensor (WCB) was developed to overcome these problems. WCB is a microorganism-based sensor that is used to detect materials [9]. Microorganisms have been used to produce biochemicals, cosmetics, pharmaceuticals, and biomaterials for industrial applications [10,11,12]. If the final products can be converted to materials capable of emitting a signal, the host microorganisms can be used as biosensors. For heavy metal detection, promoters that specifically bind to each heavy metal can be used. For a signal, the reporter gene that makes the signal is located after the promoter. Signal products of reporter genes include fluorescence and luminescence proteins. Some promoters also express a repressor that regulates the expression of the promoter. Therefore, if the target metal binds to the repressors that are present in the cell, the repressor will be released from the promoter and the promoter will be activated. In this way, the binding of the target metal to the promoter can trigger the expression of the reporter gene. Recent studies on heavy metal detection using WCBs have reported certain obstacles preventing the practical use of WCBs. These WCBs show a narrow detection range and need one or two kinds of targets and additional instruments [9,13,14,15]. To overcome these obstacles, we tested three heavy metals—copper, cadmium, and mercury, respectively—in the detection range that was selected after considering the Korea Food and Drug Administration’s Food code and a recent study [4]. In addition, we constructed visible WCBs using mCherry [7], which will help in their commercialization.

## 2. Materials and Methods

### 2.1. Bacterial Strain, Plasmid, Medium, and Heavy Metals

*Escherichia coli* DH5α [*end*A1, *rec*A1, *rel*A1, *deo*R, *gyr*A96, *hsd*R17 (r^−^k, m^+^k), *sup*E44, *thi*-1, Δ (*lac*ZYA-*arg*F) U169, φ80d*lac*ZΔM15, λ^−^, F^−^] was used as a host strain to manipulate recombinant DNA. The high-copy-number plasmid pUC57 carrying the ampicillin resistance gene was used to express the metal-sensing genes (*copA* promoter-*lux*, *zntA* promoter-*lux*, *merR*-*mer* promoter-*ompA*-*mcherry*, *copA* promoter-*ompA*-*mcherry*, *zntA* promoter-*ompA*-*mcherry*). A Luria-Bertani broth medium (LB) containing 10 g/L tryptone, 5 g/L yeast extract, and 10 g/L sodium chloride, and M9 minimal medium containing 6.78 g/L disodium phosphate, 3 g/L monopotassium phosphate, 0.5 g/L sodium chloride, 1 g/L ammonium chloride, 4 g/L glucose, 0.1 mM calcium chloride, and 2 mM magnesium sulfate was used in this study. Both media contained 50 μg/mL ampicillin. For standard solutions, chlorinated heavy metal ions (Cd^2+^, Cu^2+^, and Hg^2+^; Sigma Aldrich, St. Louis, MO, USA) were used after dissolving in distilled water.

### 2.2. Construction of Heavy Metal-Sensing Plasmids

**Figure A1:**
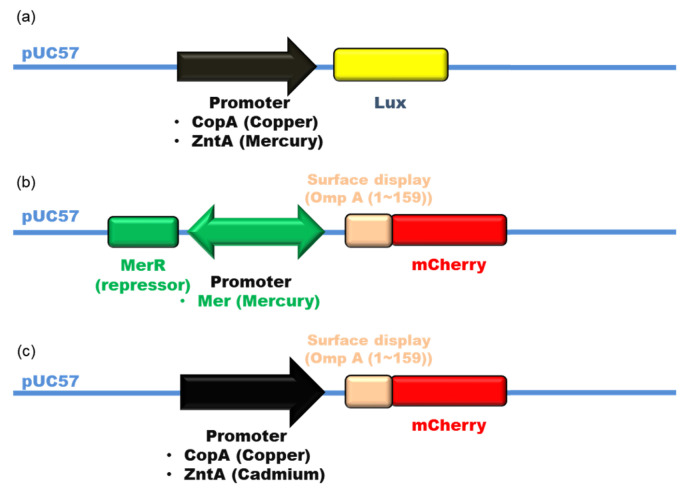
Whole metal-sensing plasmid modules. (**a**) pCopAp-Lux and pZntAp-Lux. (**b**) pMerp-mCherry. (**c**) pCopAp-mCherry and pZntAp-mCherry.

Metal-sensing plasmids were constructed using pUC57 as a template vector in the following structure. Each metal-sensing promoter, and reporter genes were synthesized and were cloned into *EcoR*I-*Hind*III restriction sites. The *lux* gene was used as a luminescent metal-sensing gene. The *copA* promoter was used for copper detection, and the *zntA* promoter was used for mercury detection. Sequence data were obtained from the Biocyc database collection (https://biocyc.org/, accessed on 5 January 2021). Using these three components, the luminescent metal-sensing vectors pCopAp-Lux for sensing copper and pZntAp-Lux for sensing mercury were constructed. For the fluorescent metal-sensing, *ompA* (1–159)-fluorescent gene module synthesized by Cosmogenetech (Seoul, Korea) was selected. The *copA*, *zntA*, and *mer* promoters were used for detecting copper, cadmium, and mercury, respectively. Additionally, residues 1–159 of the outer membrane protein OmpA were used for the surface display of the visual detectable fluorescent protein mCherry (red color). For the mercury-sensing plasmid (pMerp-mCherry), the gene of *merR*-*mer* promoter-*ompA* (1–159)-*mcherry* was synthesized and cloned into the pUC57 vector. The *mer* promoter is a bidirectional promoter derived from the Tn*501* transposon [7]. The repressor *merR* was placed before the *mer* promoter. The three protein sequences were obtained from Uniprot (https://www.uniprot.org/, accessed on 2 February 2021). For the copper-sensing plasmid (pCopAp-mCherry), the *copA* promoter-*ompA* (1–159)-*mcherry* gene module was synthesized. For the cadmium-sensing plasmid (pZntAp-mCherry), the *zntA* promoter-*ompA* (1–159)-*mcherry* gene module was synthesized [16]. Thus, three fluorescent metal-sensing plasmids, pMerp-mCherry, pCopAp-mCherry, and pZntAp-mCherry, were constructed. The whole metal-sensing plasmid modules were displayed at Figure A1.

### 2.3. Engineering of Metal-Sensing E. coli

For construction of the luminescent strains, pCopAp-Lux and pZntAp-Lux plasmids were transformed into *E. coli* by electroporation, respectively. For fluorescent metal-sensing strains, pCopAp-mCherry, pZntAp-mCherry, and pMerp-mCherry were transformed into each *E. coli*, respectively. 

### 2.4. Detection of the Three Metals 

To test the responses of the metal-sensing strains to Hg^2+^, Cu^2+^, and Cd^2+^, *E. coli* DH5α harboring luminescent and fluorescent metal-sensing, plasmids, respectively were incubated in LB media at 37 °C and 220 rpm overnight. Subsequently, the strains were diluted at 1:100 and further cultivated. The luminescent cells were incubated in M9 medium until the optical density at 600 nm (OD_600_) was equal to 1. The bacterial solution was then incubated with 5% luciferin and 10% of the corresponding metal solution (0–5 ppm) at 37 °C for 4 h. This response was conducted using 96-well plates with air porous tape. For fluorescent strains, cells in LB media were cultivated until reaching an OD_600_ of 0.5, after which they were pelleted by centrifugation (3000 rpm, 5 min), resuspended in M9 medium, and incubated at 37 °C with shaking at 220 rpm for 2–3 h. Finally, the solution was concentrated using centrifugation until reaching an OD_600_ of approximately 5. After this process, three kinds of metal solutions (copper, cadmium, and mercury) were added at different concentrations to each fluorescent metal-sensing strains. The copper-sensing strain was tested by adding copper in the range of 2–6 ppm and 0.5 ppm of other metals. The cadmium-sensing strain was tested by adding cadmium in the range of 0.2–0.75 ppm and 0.5 ppm and 5 ppm of mercury and copper, respectively. The mercury-sensing strain was tested by adding mercury in the range of 0.1–0.75 ppm and 0.5 ppm and 5 ppm of cadmium and copper, respectively. These samples were incubated at 37 °C with shaking at 250 rpm for 6 h to assess the linearity, visibility, and selectivity. For analyzing the linearity, relative light units (RLU) for luminescence and relative fluorescence units (RFU) for fluorescence were evaluated using a Cytation 5 (BioTek Instruments Inc, Winooski, VT, USA) imaging reader. RFU was measured using the emission and excitation wavelengths of 610 and 587 nm, respectively [7].

### 2.5. Immobilization of the Metal-Sensing Strain 

The fluorescent mercury-sensing strain was cultivated as described in Section 2.4. The transfer was performed at a dilution of 1:100 every 12 h. After the third transfer, the cells were cultivated until they reached 0.5 of OD_600_. The cell culture was then centrifuged and resuspended in M9 medium and cultivated at 37 °C with shaking at 220 rpm for 2–3 h. To generate a solution with a high cell density, the cells were centrifuged, supernatants were discarded, and the solution was diluted to 7.6 × 10^10^ CFU/mL with 2× M9 medium (Difco 5 × M9 minimal salt solution, 40%; glucose, 8%; 1 M magnesium sulfate, 0.4%; 1 M calcium chloride, 0.02%). This high cell density solution was mixed with the same volume of 3% low melting agarose gel solution, poured into 96-well plates, and allowed to harden at room temperature for 30 min. Mercury solutions (0–0.75 ppm) were poured into each well at a final concentration of 10%. Plates with air porous tape were incubated at 37 °C for 17–19 h, and fluorescence measurements were performed as described in Section 2.4. 

## 3. Results and Discussion

### 3.1. Construction of Metal-Sensing Strains 

**Figure 1:**
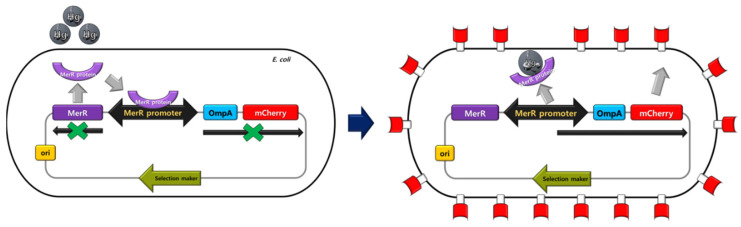
Action mechanism of the fluorescent metal-sensing strain (mercury sensing).

For heavy metal detection, *copA* (for copper) and *zntA* (for cadmium and mercury) promoters were identified in the genome of the *E. coli*. In addition, the *mer* promoter was selected from the Tn*501* transposon [17]. For signal generation, the *lux* and *mcherry* gene were selected for the luminescence signal and red color fluorescence, respectively. For enhancement of the fluorescent signal, the OmpA protein (residues 1–159) was used for the surface display of the mCherry protein [7]. In the case of luminescent signal detection, the *copA* promoter-*lux* and *zntA* promoter-*lux* gene modules were used to detect copper and mercury, respectively and the *copA* promoter-*ompA*-*mcherry*, *zntA* promoter-*ompA*-*mcherry*, and *merR*-*mer* promoter-*ompA*-*mcherry* systems were also constructed for fluorescent visible detection of copper, cadmium, and mercury gene module, respectively. The repressor genes of copper and cadmium are present in the genome of *E. coli*. However, the repressor gene of the mercury sensing promoter (*mer* promoter) is not in the genome of *E. coli*. Therefore, the *merR* gene was placed before the *mer* promoter [7]. The operation of the bidirectional *mer* promoter system in *E. coli* is shown in Figure 1 [17]. All gene modules were cloned into the pUC57 vector. For fluorescent strains, copper-, cadmium-, and mercury-sensing strains were constructed by the transformation of each plasmid containing the gene modules. In the fluorescent-sensing strains, the addition of the target metal into the cell culture shows a red color. In recent studies, most WCBs could detect only one or two kinds of metal [7,14,16]. We constructed three kinds of strains for detecting each metal specifically. These results can be useful in simplifying the process for industrial sensor production.

### 3.2. Detection of Heavy Metals by Luminescent Metal-Sensing Strains 

**Figure 2:**
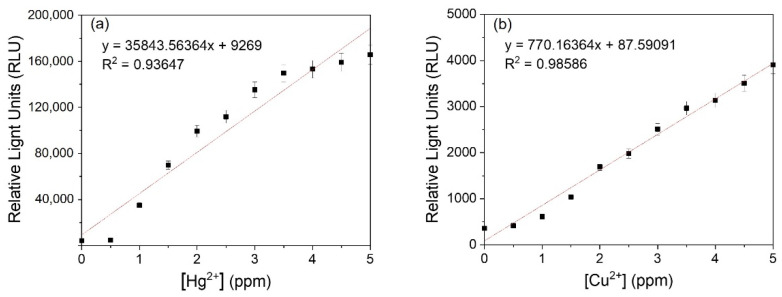
The correlations between RLUs and target metal concentration of luminescent (**a**) mercury-sensing strain and (**b**) copper-sensing strain.

The analysis of luminescent metal-sensing strains incubated with the target metal solutions for 4 h is shown in Figure 2. Studies related to WBC are usually focused on detecting metals at a low concentration [7,16]. In this study, the minimum detectable concentration of copper and mercury were 0.5 ppm. Heavy metals at this concentration cause toxicity in humans [4]. This sensor system is developed for public use and compatibility with existing instruments. Therefore, we plotted these data linearly for intuitive recognition. The linearity values for copper and mercury were 0.98586 and 0.93647, respectively, indicating that the system could accurately detect these metals within a practical concentration range. The average RLU of the mercury-sensing strain (108,324 ± 1797.4 RLU) was higher than that of the copper-sensing strain (2374 ± 38.2 RLU). This may be because the CopA protein in *E. coli* exports copper from the cytoplasm to outside of the cells [18], resulting in a reduced reaction with the copper-sensing plasmid. If the *copA* gene can be deleted, the expression of the reporter gene will be increased. This luminescent system cannot be detected without an instrument. Next, the luciferase luminescent system was changed into a fluorescent system for direct detection with the naked eye. In order to be visibly detectable, the *lux* gene was replaced with the *mcherry* gene, a red color protein. 

### 3.3. Detection of Heavy Metals by Fluorescent Metal-Sensing Strains

**Figure 3:**
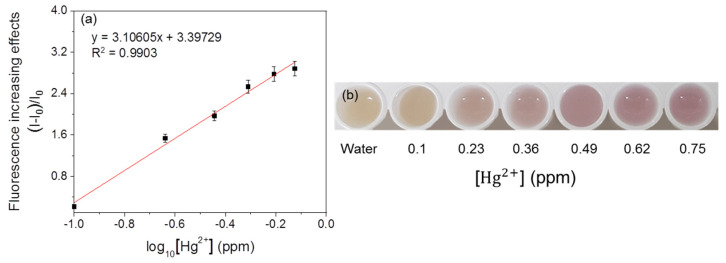
Concentration-dependent responses of fluorescent mercury-sensing strain with mercury. (**a**) The correlation between fluorescence increasing effects and mercury concentration of the strain. (**b**) Visual detection of mercury by the strain.

**Figure 4:**
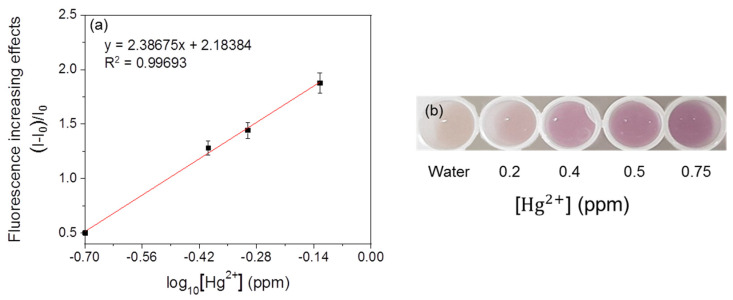
Fluorescent mercury-sensing strain after incubation in M9 medium for 3 h. (**a**) linearity and (**b**) visualization of the fluorescent signal.

**Figure 5:**
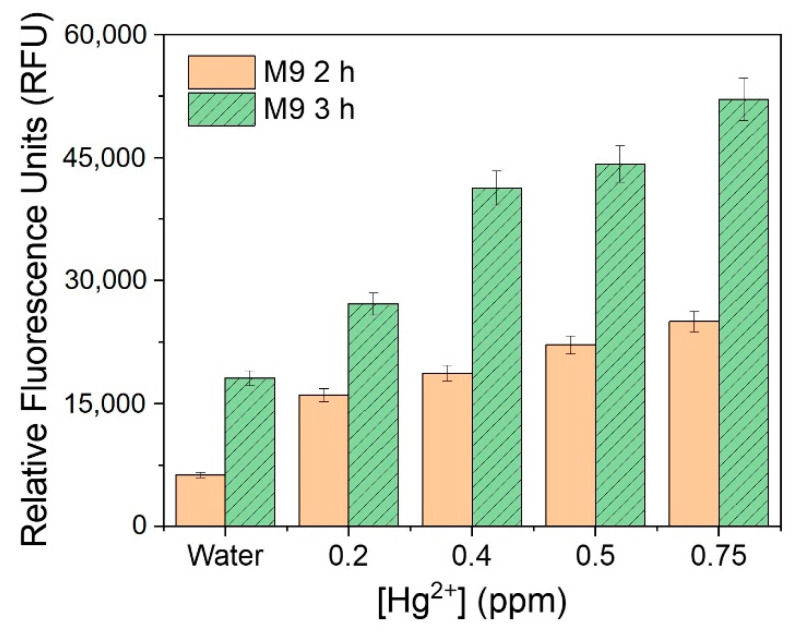
Comparison of relative fluorescence units value according to incubation time in the M9 media.

**Figure 6:**
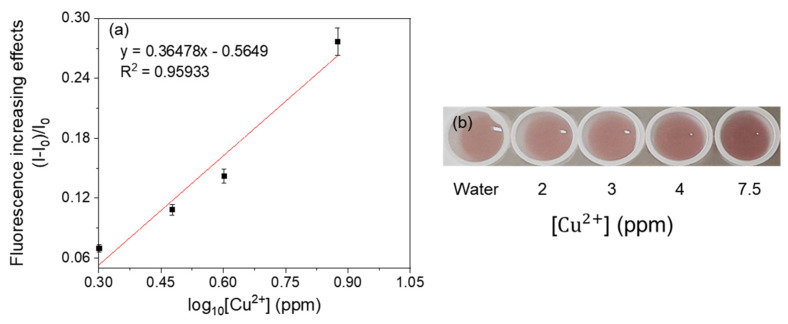
Concentration-dependent responses of fluorescent copper-sensing strain with copper. (**a**) The correlation between fluorescence increasing effects and copper concentration of the strain. (**b**) Visual detection of copper by the strain.

**Figure 7:**
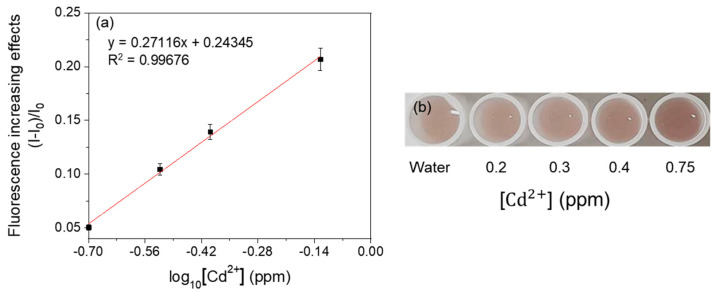
Concentration-dependent responses of fluorescent cadmium-sensing strain with cadmium. (**a**) The correlation between fluorescence increasing effects and cadmium concentration of the strain. (**b**) Visual detection of cadmium by the strain.

For linearity analysis, the fluorescence increasing effects (I–I_0_)/I_0_ (where I = RFU with metal, I_0_ = RFU with water) were assessed and plotted against the log_10_ concentration of heavy metals [7]. The linearity of the mercury-sensing strain is shown in Figure 3, and the R^2^ value obtained from this analysis was 0.99030 in the range of 0.1–0.75 ppm. As the amount of signal protein changes according to the cell number, the change in color could be directly observed. The increase in the cell incubation time from 2 h to 3 h resulted in an increase in the average RFU (Figure 4 and Figure 5), and the visibility was also increased. Consistent with this, the R^2^ value also increased compared with that after incubation for 2 h. These results could be explained by increased cell growth and the stability of the fluorescent protein. Indeed, when cells are grown in the M9 medium, the RFU increases because of the stability of the fluorescent protein [19]. Because measurements are taken during the exponential growth phase, the cell density and fluorescent activity continue to increase, making the reaction with the sample more dramatic. The colorimetric change upon different concentrations of the metal was clear (Figure 4b), similar to incubation for 2 h, as described above. Considering the activation time under the M9 medium, the results for the copper- and cadmium-sensing strain were obtained after incubation in the M9 medium for 3 h (Figure 6 and Figure 7). The R^2^ values of copper was 0.95933 in the range of 2–6 ppm. The cadmium was 0.99676 in the range of 0.2–0.75 ppm. Both copper and cadmium sensing were visible by the naked eye, and color intensity at different concentrations could be clearly distinguished. However, more detection points are needed to provide accurate data. To increase the efficiency of signaling, optimization of the M9 media components is required [20]. The lower fluorescence increasing effects in the copper- and cadmium-sensing strain than the mercury-sensing strain might be caused due to the high signal of the blank. But difference of signal intensity can be distinguished. Thus, these three fluorescent metal-sensing strains could be used to detect hazardous heavy metals at concentration ranges that could have practical uses. Most existing WBC developments need a complicated instrument for detecting fluorescence [21]. From the production process perspective, our colorimetric biosensor system is easier to use because of its simple and visible detection platform.

### 3.4. Selectivity of Fluorescent Metal-Sensing Strains

**Figure 8:**
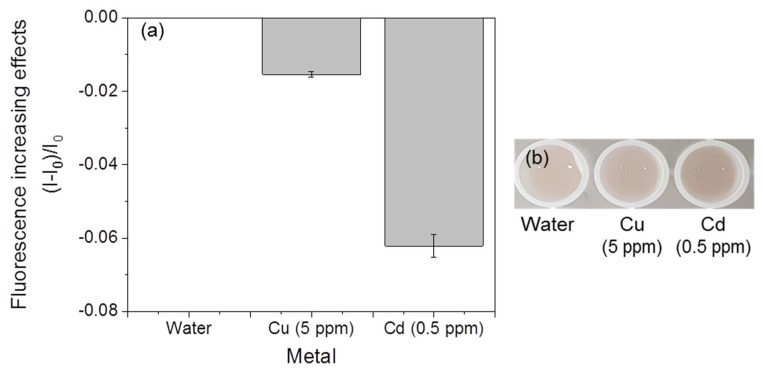
Responses of the fluorescent mercury-sensing strain with other metals. (**a**) Fluorescence increasing effects. (**b**) Visual detection of the strain.

**Figure 9:**
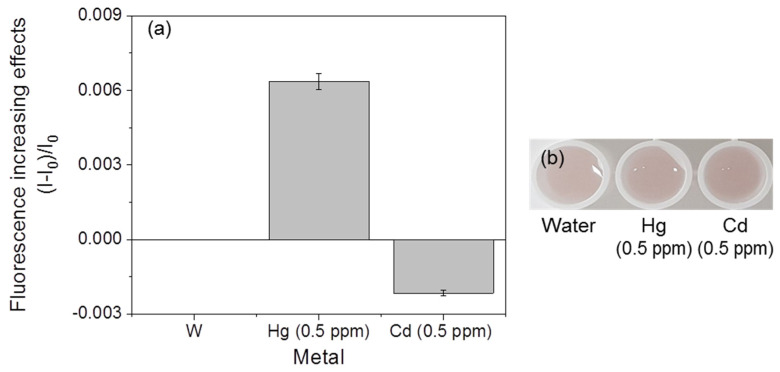
Responses of the fluorescent copper-sensing strain with other metals. (**a**) Fluorescence increasing effects. (**b**) Visual detection of the strain.

**Figure 10:**
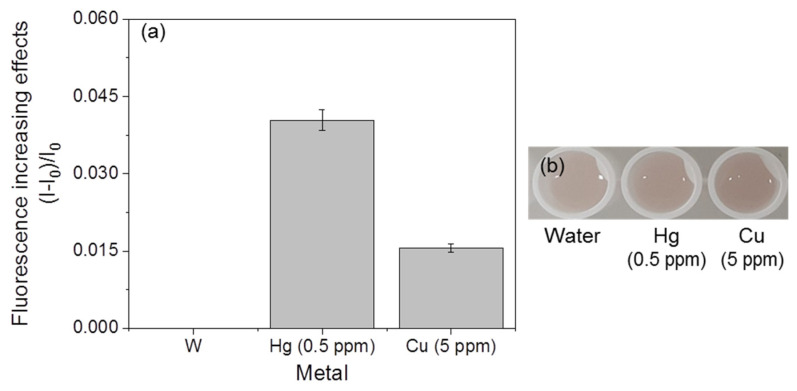
Reaction of the fluorescent cadmium-sensing strain with other metals. (**a**) Fluorescent increasing effect. (**b**) visualization of the fluorescent signal.

The mercury-sensing strain did not show fluorescent responses, and significant color changes were not observed in the presence of cadmium and copper (Figure 8). However, the responses of the copper-sensing strain with other metals, such as mercury and cadmium, were observed (Figure 9). A slight increase (0.006) in the fluorescence increasing effect was detected in response to mercury, but this value was much lower than the values of the mercury-sensing strain’s response to mercury (0.05–0.28, Figure 9a), and no significant color changes were observed (Figure 9b). The cadmium sensor responded to mercury and copper (Figure 10). However, the values of these responses were lower than the lowest value of the mercury-sensing and copper-sensing strain’s response with responsible metal (Figure 3a and Figure 6a). Additionally, no significant color changes were observed with water (Figure 10b). The fluorescent cadmium-sensing strain was unable to be accurately distinguished. For increased the accuracy, deletion of *zntR*, which is a repressor protein for the *zntA* promoter, and the insertion of a gene sequence expressing a cadmium-response repressor to the cadmium-sensing plasmid can be the solution [16]. This would decrease the slight response to mercury. Each strain showed a visible and distinguishable color change response to its specific corresponding heavy metal; therefore, these strains could be used within a kit to detect the three metals. 

### 3.5. Immobilization of the Fluorescent Metal-Sensing Strain

**Figure 11:**
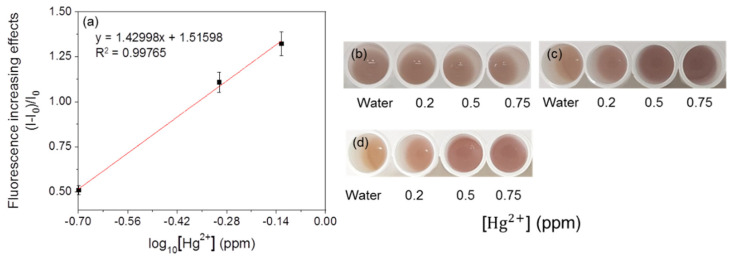
Concentration-dependent responses of the immobilized fluorescent mercury-sensing strain with mercury. (**a**) The correlation between fluorescence increasing effects and mercury concentration of the strain. Visual detection of responses (**b**) before responses, (**c**) after 17 h, and (**d**) 19 h.

**Figure 12:**
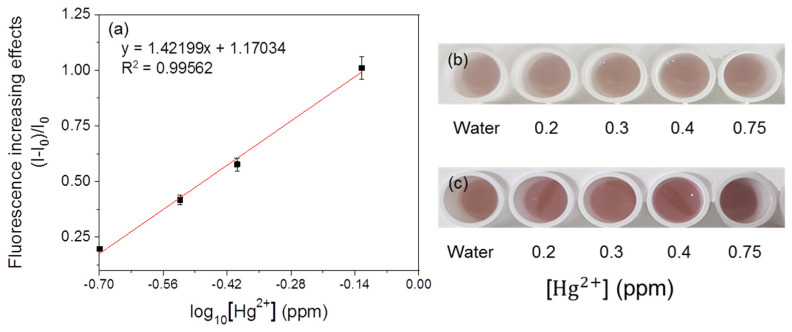
Effects of 3 h incubation in M9 medium on fluorescent value (RFU). (**a**) The correlation between fluorescence increasing effects and mercury concentration of the immobilized strain. Visual detection of responses (**b**) before responses and (**c**) after 19 h.

**Figure A2:**
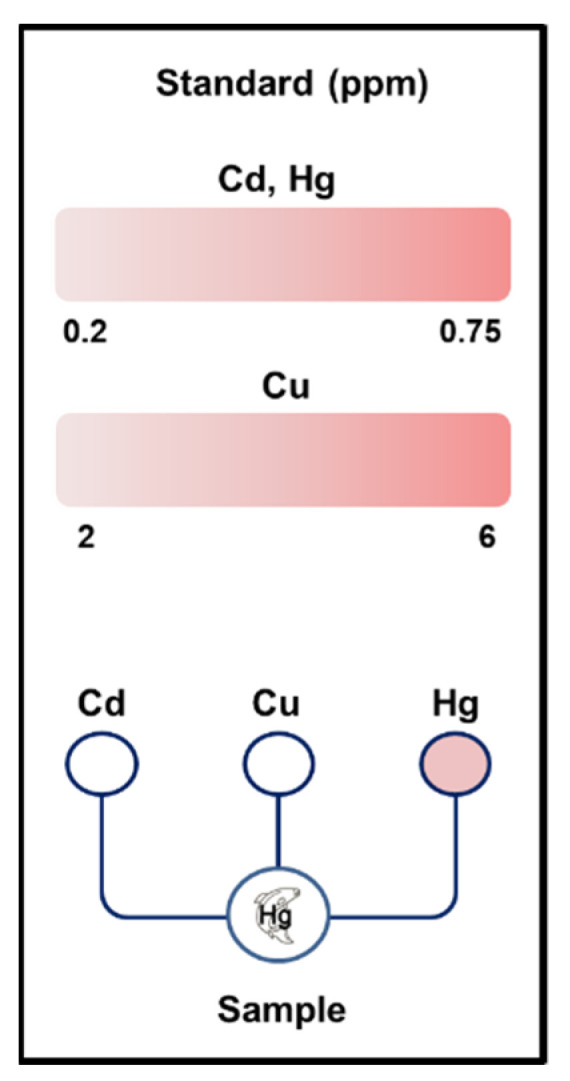
Product design of colorimetric metal sensor.

The results of sensitivity in the immobilized mercury-sensing strain are shown in Figure 11. The linearity was 0.99765, but the color responses could be recognized after 17 h. At this time, the color changes in response to different concentrations of mercury could be distinguished. After 19 h, the differences were clearly visible. Similar to the experiment in M9 liquid media, increasing incubation time resulted in a brighter color in the M9 solid media (Figure 12) and the linearity value was 0.99562 (Figure 12a). This result suggests that strains in this colorimetric detection system could survive under these conditions [22]. However, the time required to observe the response visibly was increased, and the average efficiency of this immobilization is 46% which is calculated by comparing fluorescence increasing effects. Immobilization would make the mass transfer within the culture environment much slower and would retard the bacterial growth [23]. This effect may be overcome by changing the immobilization material to create a more porous environment [24] or the immobilization of high-density cells. Additionally, immobilization with agarose could overcome the problems related to shaking in the liquid and is better suited for user-friendly sensors that can be easily transported and moved [7]. In this system, a multiplexing system detecting three kinds of metals can be developed (Figure A2). For increasing the accuracy and reliability of this system, various tests, including the immobilization of other strains, will be required. 

## 4. Conclusions

In this study, WCBs that are user-friendly, low-cost, and effective were developed. Currently available WCBs have various limitations, such as a low detection range, which is not suitable for the detection of toxic concentrations to humans, detection of only one or two targets, and the necessity for extra expensive instruments or equipment. Using these WCBs, metals in the concertation range that is toxic to humans could be detected easily using luminescent metal-sensing strains; this range also covered the concentrations considered safe in edible fish in Korea, suggesting that these WCBs could be used by fishery workers. Moreover, the WCBs could selectively detect copper, cadmium, and mercury and may be expanded to detect additional metals. Visualization of the response by the naked eye was enabled by replacing the *lux* gene with *mCherry* as a reporter gene, which shows detectable red fluorescence. Increasing the M9 incubation time was shown to further increase the intensity of the signal, enhancing the RFU and brightness. Finally, the immobilization of the sensor was achieved to make the cells safe and easy to transport, thereby improving the practical application of the sensor. As a result, copper-, mercury-, and cadmium-sensing *E. coli* strains for which signals could be detected by the naked eye were obtained. Thus, the WCBs produced herein were inexpensive and user-friendly and could be used to visually detect three heavy metals, thereby providing a practical product design with potential use for fishery workers.

## Data Availability

The data presented in this study are available on request from the corresponding author.
